# The Pathologically Evolving Aggregation-State of Cells in Cancerous Tissues as Interpreted by Fractal and Multi-Fractal Dispersion Theory in Saturated Porous Formations

**DOI:** 10.3390/bioengineering11050469

**Published:** 2024-05-08

**Authors:** Marilena Pannone

**Affiliations:** School of Engineering, University of Basilicata, 85100 Potenza, Italy; marilena.pannone@unibas.it

**Keywords:** biological tissue architecture, diffusion-limited fractal dimension, stochastic solute transport models, cancerous tissue mapping

## Abstract

A recent author’s fractal fluid-dynamic dispersion theory in porous media has focused on the derivation of the associated nonergodic (or effective) macrodispersion coefficients by a 3-D stochastic Lagrangian approach. As shown by the present study, the Fickian (i.e., the asymptotic constant) component of a properly normalized version of these coefficients exhibits a clearly detectable minimum in correspondence with the same fractal dimension (*d* ≅ 1.7) that seems to characterize the diffusion-limited aggregation state of cells in advanced stages of cancerous lesion progression. That circumstance suggests that such a critical fractal dimension, which is also reminiscent of the colloidal state of solutions (and may therefore identify the microscale architecture of both living and non-living two-phase systems in state transition conditions) may actually represent a sort of universal nature imprint. Additionally, it suggests that the closed-form analytical solution that was provided for the effective macrodispersion coefficients in fractal porous media may be a reliable candidate as a physically-based descriptor of blood perfusion dynamics in healthy as well as cancerous tissues. In order to evaluate the biological meaningfulness of this specific fluid-dynamic parameter, a preliminary validation is performed by comparison with the results of imaging-based clinical surveys. Moreover, a multifractal extension of the theory is proposed and discussed in view of a perspective interpretative diagnostic utilization.

## 1. Introduction

As shown by recent studies (e.g., [1,2]), oncogenesis is associated with the pathologic evolution of cellular (and, as a consequence, microvascular) clustering, driven by leader-cell inter-cluster migration and progressive empty space filling. Indeed, as the cancerous cell proliferation proceeds, the microvascular bed experiences a similar morphological evolution, targeted to the feeding of the new cells by repeated branching. This process proves to be responsible for the gradual increase in tissue fractal dimension (1.5<d<1.6 the healthy value) up to about 1.7. In a nutshell, one can say that fractality is a synonym of self-similarity, i.e., of the systematic replication of a given deterministic or random structure at all (or at a part of) the associated spatial or temporal scales. In the case of spatial distributions, the fractal dimension may be defined as a global index of their geometrical complexity and, ultimately, as a measure of the capability of the given pattern to fill the space.

A recent author’s theoretical investigation ([3]) focused on fluid-dynamic dispersion in fractal geologic formations by a 3-D first-order stochastic Lagrangian approach according to [4,5]. In this study, effective (or nonergodic) macrodispersion coefficients were derived as the half time-rate of change of the expected central inertia moments of a tracer plume that originates from a point solute pulse. The fully analytical treatment, which was corroborated by comparison with field survey observations, interpreted medium isotropic fractal log-conductivity Y=lnK as a double continuous hierarchy of mutually independent stationary random fields. All the derivations were performed in dimensionless terms by subdividing the associated unbounded frequency domain into a medium/high-frequency core and a low-frequency tail and by adopting the inverse of the boundary frequency *k*_0_ as the reference geometric scale. The ensemble-mean velocity magnitude U was used as the kinematic scale.

As is well known, a stationary random distribution is identified by a single scale of heterogeneity (e.g., [6]). The hierarchical log-conductivity field medium/high-frequency core (which contributes to fluid macrodispersion with the medium/smaller scales of the heterogeneity) yielded large-time constant, Fickian-like macrodispersion coefficients; the low-frequency tail (which represents the larger scales of the heterogeneity) was responsible for the time-increasing, “anomalous” counterparts.

When applied to fluid-dynamic dispersion in biological tissues, porous media flow and transport theory (see [7] for a discussion about the soundness of such an approach to the investigation of the related processes), which was already successfully applied by the author to ventricle wall perfusion by a single-scale deterministic approach ([8]), must refer to an upper-bounded sequence of heterogeneity scales even in the presence of fractal-like architectures. As a matter of fact, the existence of an intrinsic upper limit of tissue (and micro-vessels) self-similar geometry is implied by the necessarily finite largest scale of the pre-existing cell clusters. Hence, the perfusion-related dispersion of solutes in biological tissues may consistently be modelled and investigated by the above-described Fickian component of the macrodispersion coefficients in fractal porous media, as arising from the heterogeneity scales that are smaller than the typical cell cluster dimension. Note that, in the hydrogeologic analogy, cells, extracellular matrix and blood vessel walls are functionally represented by the sedimentary skeleton, while blood has to be thought of as flowing through the more or less tortuous canaliculi formed by the connected pores.

The present investigation was inspired by the author’s finding that the pathologic, diffusion-limited fractal dimension that characterizes most late-stage cancerous tissues was exactly the same as the one that identified the minimum of both the longitudinal and the transverse normalized macrodispersion coefficient in fractal, anti-persistently correlated geologic formations. Its first objective is to demonstrate the biological meaningfulness of such fluid-dynamic parameters. The second is represented by a multi-fractal extension of the macrodispersion theory that may encompass dispersion coefficients spatial heterogeneity, and may therefore allow for an indirect estimation of tissue fractal dimension variability (or, in other words, of tumor progression stage).

The rest of the manuscript is organized as follows: In the Section 2, the fundamental steps for the derivation of the normalized macrodispersion coefficients are outlined according to [3], along with the needed extension in terms of log-conductivity and related velocity spectra in the case of multi-fractality. Note that such multi-fractality has to be intended as a locally simply fractal log-conductivity distribution with a superposed slow deterministic fractal dimension trend. In the Section 3, a preliminary validation of the simply fractal approach is proposed by comparing perfusion-related dispersion coefficients obtained from imaging-based clinical surveys, available in the literature and related to a single-value estimation for the whole tissue, to the theoretical predictions. Additionally, the multi-fractal extension of the theory is operatively described and discussed by resorting to the detection cell-scale numerical Lagrangian simulation of the heterogeneously diffusive transport (and the associated dispersion coefficient evaluation procedure). Finally, the Section 4 summarizes the main results, framing them in a more general natural context, and proposes possible further steps of the research that may lead to routine clinical applications.

## 2. Materials and Methods

In the vast majority of cases, the detailed deterministic analytical description of flow and transport processes taking place within saturated natural porous structures is prevented by their typically marked heterogeneity. In the last few decades of the twentieth century, the stochastic approach to the problem, in both the Eulerian and the Lagrangian formulation, became very popular as a useful and reasonably affordable mathematical tool for its solution, at least in terms of first statistical moments of the involved variables. An exhaustive review of the great quantity of valuable studies that it has been producing since then would practically be impossible. Later on, the specific investigations to which the key results that constitute the starting points of the present mathematical treatment are due will be referenced where appropriate.

The most common stochastic models of subsurface flow and transport assume that the space-dependent medium log-conductivity (i.e., the natural logarithm of the hydraulic conductivity of the ensemble porous matrix/permeating fluid) is a statistically stationary and normally distributed random variable. As mentioned in the Introduction, the stationary assumption implies the existence of a single scale of the heterogeneity. However, in several cases, the experimental evidence suggested the possibility that the porous matrix exhibited a self-similar organization, i.e., that it reproduced itself over all the involved physical scales (no frequency cutoff) or, more likely, over a part of them (lower and/or upper frequency cutoff).

The outcome of recent clinical investigations seems to authorize the assumption that the micro-structure of many biological tissues (typical multi-phase systems) also exhibits a self-similar organization, which can pathologically evolve from basic, relatively empty clusters to dense aggregates of cells. The scope of the present section is to synthetically illustrate the mathematical formulation, borrowed from hydrogeology and geostatistics, that may help model tissue perfusion-related dispersion in the presence of micro-scale fractal structures represented by a uniform or a space-dependent aggregation degree.

A statistically stationary space-dependent random function (in the case under investigation, the hydraulic log-conductivity $Y\left(x\right)=\ln K\left(x\right)$) is by definition represented by a constant ensemble mean $\langle Y\rangle =\langle Y\left(x\right)\rangle$, a covariance function that just depends (typically by a negative exponential or a Gaussian law) on the distance between the points whose degree of correlation is being evaluated (e.g., [6]):

(1) $$R_{Y}=R_{Y}\left(r\right)=\langle Y^{\prime }\left(x\right)Y^{\prime }\left(x+r\right)\rangle$$

and by a constant variance $\sigma_{Y}^{2}=R_{Y}\left(0\right)$. In the above formulas and in what follows, angle brackets $\langle \cdot \rangle$ identify ensemble averaging and the prime indicates the deviation about the mean: $Y^{\prime }\left(x\right)=Y\left(x\right)-\langle Y\rangle$. In the isotropic case, the correlation functions depend on the magnitude of the vector distance $r=\left|r\right|$ only. The semi-variogram represents the (half) variance of the spatial increments of the given function (in this case Y) (e.g., [6]):

(2) $$\gamma_{Y}\left(r\right)=\frac{1}{2}{\langle {\left[Y\left(x+r\right)-Y\left(x\right)\right]}^{2}\rangle }_{r=\left|r\right|}=\sigma_{Y}^{2}-R_{Y}\left(r\right)$$

and tends to $\sigma_{Y}^{2}$ when *r* approaches infinity, at a rate that depends on the magnitude of the correlation length.

Fully evolving-scale or fractal random structures (e.g., [9]) exhibit self-similarity over all scales of heterogeneity, which prevents the asymptotic stabilization of the semi-variogram and makes it behave like a power law. For values of the related scaling exponent *b* that range between 0 and 1 (extremes excluded), i.e., for a less than linearly increasing semi-variogram, the given random evolving-scale distribution is said to be characterized by “anti-persistent” correlations; for 1 ≤ *b* < 2, i.e., for a linearly or more than linearly increasing semi-variogram, it is said to be characterized by “persistent” correlations (e.g., [10]). The normalized large-time Fickian longitudinal (D_mL_) and transverse (D_mT_) macrodispersion coefficient derived by [3] for ensemble mean velocity $U=\left(U,0,0\right)$, and here reported as a function of the fractal dimension *d* = 2 − *b*/2 for 0<b<1, reads:

(3) $$D_{mL}=\frac{D_{mL}k_{0}}{U}=\frac{B\left(\frac{7-2d}{2},\frac{2d-3}{2}\right)}{\left(6-2d\right)4^{\left(5-2d\right)/2}\pi^{5-2d}}\left(\frac{1}{2}-\frac{20}{3Pe}\right)$$

(4) $$D_{mT}=\frac{D_{mT}k_{0}}{U}=\frac{B\left(\frac{7-2d}{2},\frac{2d-3}{2}\right)}{3\left(6-2d\right)4^{\left(5-2d\right)/2}\pi^{5-2d}Pe}$$

In Equations (3) and (4), italics indicate the dimensional coefficients, *Pe* = *U*/*k*_0_*D*_0_ the typically very large Péclet number (a fluid-mechanical measure of the relative importance of advective and purely diffusive transport mechanisms), *D*_0_ the pure diffusion coefficient, and B the Beta function (e.g., [11]):

(5) $$B\left(\alpha ,\beta \right)=\int_{0}^{1}\xi^{\alpha -1}{\left(1-\xi \right)}^{\beta -1}d\xi$$

The hierarchical log-conductivity semi-variogram was obtained from the following linear combination:

(6) $${\tilde{\gamma }}_{Y}\left(r\right)=ar^{b}=\int_{0}^{\infty }\frac{\partial }{\partial \lambda }\left[\gamma_{Y}\left(r,\lambda \right)\right]d\lambda =\int_{0}^{\infty }\frac{\phi }{\lambda^{1+b}}\left[1-\exp\left(-r\lambda \right)\right]d\lambda \;\;\;0<b<1$$

In Equation (6), ${\gamma_{Y}\left(r,\lambda \right)=\sigma }_{Y}^{2}\left(\lambda \right)-R_{Y}\left(r,\lambda \right)$ indicates the stationary, primary-hierarchy single-component semi-variogram; $\sigma_{Y}^{2}\left(\lambda \right)$ and $R_{Y}\left(r,\lambda \right)$ respectively indicate the stationary, primary-hierarchy single-component log-conductivity variance and covariance; ϕ is a dimensional constant, and *λ* = 1/*I_Yλ_* is the inverse of the single-component integral scale (or correlation length). Additionally, here and in what follows, the tilde accent indicates quantities that are obtained by the linear combination of the single-scale counterparts. Note that, for 0<b<1, the scaling exponent and the scaling coefficient are related by:

(7) $$a=-\phi \Gamma \left(-b\right)=-k_{0}^{b}\Gamma \left(-b\right)$$

with Γ indicating the Gamma function (e.g., [11]):

(8) $$\Gamma \left(\alpha \right)=\int_{0}^{\infty }\exp\left(-\xi \right){\left(1-\xi \right)}^{\alpha -1}d\xi$$

The Lagrangian derivation of the nonergodic macrodispersion coefficients in Equations (3) and (4) starts by recognizing that for stationary log-conductivity distributions and solute point pulses (see [5] for the details):

(9) $$D_{mii}\left(t\right)=\frac{1}{2}\frac{d\langle I_{ii}\rangle }{dt}=\frac{1}{2}\frac{dX_{ii}}{dt}-\frac{1}{2}\frac{d\Theta_{ii}}{dt}=D_{miiE}\left(t\right)-\frac{1}{2}\frac{d\Theta_{ii}}{dt}\;\;\;i=1,2,3$$

where $\langle I_{ii}\rangle$ indicates the *i*th expected central inertia moment, and $X_{ii}$ and $\Theta_{ii}$ the *i*th one- and two-particle trajectory variance/covariance, respectively:

(10) $$X_{ii}\left(t\right)=\langle X_{i}^{\prime 2}\left(t\right)\rangle =\langle {\left(X_{i}\left(t\right)-\langle X_{i}\left(t\right)\rangle \right)}^{2}\rangle =\langle {\left(X_{i}\left(t\right)-U_{i}t\right)}^{2}\rangle$$

(11) $$\Theta_{ii}\left(t\right)=\langle X_{i}^{\prime }\left(t\right)Z_{i}^{\prime }\left(t\right)\rangle =\langle \left(X_{i}\left(t\right)-\langle X_{i}\left(t\right)\rangle \right)\left(Z_{i}\left(t\right)-\langle Z_{i}\left(t\right)\rangle \right)\rangle \;=\langle \left(X_{i}\left(t\right)-U_{i}t\right)\left(Z_{i}\left(t\right)-U_{i}t\right)\rangle$$

$X=X\left(t\right)$ and $Z=Z\left(t\right)$ are two different trajectories, and $D_{miiE}$ is the ergodic component of $D_{mii}$ (which coincides with the half time-rate of change of $X_{ii}$). It is worth noting that, in the present context, the rather general concept of ergodicity (e.g., [12]) has to do with the possibility of considering particle dispersion evaluated from a single trajectory over the ensemble of possible flow field realizations as coinciding with particle dispersion evaluated from an ensemble of trajectories in a single flow field realization (see [13] for a discussion about the implications of the ergodic hypothesis in subsurface solute transport analysed in a Lagrangian framework).

The generic particle trajectory is represented by the following integral-differential equation:

(12) $$X\left(t\right)=\int_{0}^{t}u\left[X\left(s\right)\right]ds=Ut+X^{\prime }\left(t\right)+X_{B}\left(t\right)$$

where **u** is the steady actual velocity

(13) $$u\left(x\right)=U+u^{\prime }\left(x\right)$$

$X_{B}$ is the pure diffusion-related Brownian component, and

(14) $$X^{\prime }\left(t\right)=\int_{0}^{t}u^{\prime }\left[X\left(s\right)\right]ds$$

is the advective deviation about the mean. Thus,

(15) $$\frac{dX_{ii}}{dt}=\langle 2X_{i}^{\prime }\frac{dX_{i}^{\prime }}{dt}\rangle +2D_{0ii}=2\int_{0}^{t}\langle u_{i}^{\prime }\left[X\left(s\right)\right]u_{i}^{\prime }\left[X\left(t\right)\right]\rangle ds+2D_{0ii}=2\int_{0}^{t}R_{uii}\left[X\left(t\right)-X\left(s\right)\right]ds+2D_{0ii}$$

and

(16) $$\frac{d\Theta_{ii}}{dt}=\langle Z_{i}^{\prime }\frac{dX_{i}^{\prime }}{dt}\rangle +\langle X_{i}^{\prime }\frac{dZ_{i}^{\prime }}{dt}\rangle =\int_{0}^{t}\langle u_{i}^{\prime }\left[Z\left(s\right)\right]u_{i}^{\prime }\left[X\left(t\right)\right]\rangle ds+\int_{0}^{t}\langle u_{i}^{\prime }\left[X\left(s\right)\right]u_{i}^{\prime }\left[Z\left(t\right)\right]\rangle ds=2\int_{0}^{t}R_{uii}\left[X\left(t\right)-Z\left(s\right)\right]ds$$

where $R_{uii}$ indicates the *i*th stationary velocity covariance function, and the Brownian components of two different trajectories are uncorrelated by definition. The first-order (linearized) combination of Darcy’s law (e.g., [14]):

(17) $$u\left(x\right)=-\frac{K\left(x\right)}{\eta }\nabla h\left(x\right)$$

and continuity:

(18) $$\nabla \cdot u\left(x\right)=0$$

that is:

(19) $$\nabla^{2}h\left(x\right)+\nabla Y\left(x\right)\cdot \nabla h\left(x\right)≅\nabla^{2}h^{\prime }\left(x\right)-J\cdot \nabla Y^{\prime }\left(x\right)=0$$

where $K=\exp\left(Y\right)$ indicates the hydraulic conductivity, *h* the hydraulic head, $J=-\nabla \langle h\left(x\right)\rangle$ the mean head gradient, *η* medium porosity, and only the terms that are proportional to the first power of the deviations are retained, allows each $R_{uii}\left(r,\lambda \right)=\langle u_{i}^{\prime }\left(x;\lambda \right)u_{i}^{\prime }\left(x+r;\lambda \right)\rangle$ to relate to each $R_{Y}\left(r,\lambda \right)$ appearing in Equation (6). Note that, by virtue of the superposition principle, the linear combination of stationary covariances and semi-variograms as expressed by Equation (6) is still representative of a stationary statistical distribution. As a consequence, and in order to solve Equations (15) and (16) in the presence of hierarchical media, the needed log-conductivity covariance was obtained from the integration of the single-scale exponential component over the whole hierarchy of scales according to Equation (6):

(20) $${\tilde{R}}_{Y}\left(r\right)=\int_{0}^{\infty }\frac{\partial }{\partial \lambda }\left[R_{Y}\left(r,\lambda \right)\right]d\lambda =\int_{0}^{\infty }\frac{\phi }{\lambda^{1+b}}\exp\left(-r\lambda \right)d\lambda \;\;\;0<b<1$$

The spectral representation of the hierarchical velocity covariance came from a straightforward generalization of the single-scale counterpart according to [3]:

(21) $${\tilde{R}}_{uii}\left(r\right)=\left\langle {\tilde{u}}_{i}^{\prime }\left(x\right){\tilde{u}}_{i}^{\prime }\left(x+r\right)\right\rangle =\int_{k}{\tilde{S}}_{uii}\left(k\right)\exp\left(j2\pi k\cdot r\right)dk$$

where ${\tilde{S}}_{uii}\left(k\right)$ indicates the *i*th hierarchical velocity spectrum. The relationship between the velocity spectrum ${\tilde{S}}_{uii}\left(k\right)$ and the log-conductivity spectrum ${\tilde{S}}_{Y}\left(k\right)$ was derived from the hierarchical version of the stationary, single-scale spectral solution of Equation (19) for $U=\left(U,0,0\right)$ as reported by [14,15]:

(22) $${\tilde{S}}_{uii}\left(k\right)=U^{2}{\left(\delta_{i1}-\frac{k_{1}k_{i}}{\left|k\right|}\right)}^{2}{\tilde{S}}_{Y}\left(k\right)$$

where $\delta_{ij}$ indicates Kronecker’s Delta and $k=\left|k\right|$ the wave-number vector magnitude.

The integral-differential form of the generic hierarchical nonergodic macrodispersion coefficient then reads:

(23) $$D_{mii}\left(t\right)=\frac{1}{2}\frac{d{\tilde{X}}_{ii}}{dt}-\frac{1}{2}\frac{d{\tilde{\Theta }}_{ii}}{dt}=\int_{0}^{t}\left\{{\tilde{R}}_{uii}\left[\tilde{X}\left(t\right)-\tilde{X}\left(s\right)\right]-{\tilde{R}}_{uii}\left[\tilde{X}\left(t\right)-\tilde{Z}\left(s\right)\right]\right\}ds+D_{0ii}$$

Starting from Equation (23), reducing the argument of the velocity covariances to:

(24) $$\tilde{X}\left(t\right)-\tilde{X}\left(s\right)≅U\left(t-s\right)+X_{B}\left(t\right)-X_{B}\left(s\right)$$

and

(25) $$\tilde{X}\left(t\right)-\tilde{Z}\left(s\right)≅U\left(t-s\right)+X_{B}\left(t\right)-Z_{B}\left(s\right)$$

in order to consistently linearize the integral-differential equation, and assuming the Brownian trajectories as characterized by independent normal distributions (with $D_{0ii}$ invariably equal to $D_{0}$), led to the following general result, here expressed in a spherical reference system:

(26) $$D_{mii}\left(t\right)=\int_{k_{0}}^{\infty }\int_{0}^{\pi }\int_{0}^{2\pi }\frac{{\tilde{S}}_{uii}\left(k,\theta ,\varphi \right)4\pi^{2}k^{2}\left[1+\exp\left(-8\pi^{2}D_{0}k^{2}t\right)-2\cos\left(2\pi kU\sin\theta \cos\varphi t\right)\exp\left(-4\pi^{2}D_{0}k^{2}t\right)\right]}{{\left(2\pi kU\sin\theta \cos\varphi \right)}^{2}+16\pi^{4}D_{0}^{2}k^{4}}\;k\sin\theta d\theta d\varphi dk$$

In Equation (26), *θ* is the polar angle, *φ* the azimuthal angle, and the typically very small additive pure-diffusion contribution expressed by $D_{0}$ was neglected. The analytical details of the derivation of the asymptotic Equations (3) and (4) starting from Equation (26) can be found in the open-access publication [3].

In the case of non-stationary distributions associated with the space-dependent scaling exponent and fractal dimension, and assuming that this dependence consists of a slow (at the detection-cell scale) deterministic trend, Equation (19) can be solved by local spectral methods for each hierarchical component. All the involved correlation functions have to be intended as conditional on the specific detection cell coordinate $X_{n}=nl$ (where **n** indicates a vector of integers and *l* the detection grid spacing). The locally stationary version of Equation (22) then reads:

(27) $${\tilde{S}}_{uii}\left(k|X_{n}\right)=U^{2}{\left(\delta_{i1}-\frac{k_{1}k_{i}}{\left|k\right|}\right)}^{2}{\tilde{S}}_{Y}\left(k|X_{n}\right)$$

with

(28) $${\tilde{S}}_{uii}\left(k|X_{n}\right)=\int_{r}{\tilde{R}}_{uii}\left(r|X_{n}\right)\exp\left(-j2\pi k\cdot r\right)dr$$

(29) $${\tilde{S}}_{Y}\left(k|X_{n}\right)=\int_{r}{\tilde{R}}_{Y}\left(r|X_{n}\right)\exp\left(-j2\pi k\cdot r\right)dr$$

(30) $${\tilde{R}}_{uii}\left(r|X_{n}\right)=\left\langle {\tilde{u}}_{i}^{\prime }\left(x\right){\tilde{u}}_{i}^{\prime }\left(x+r\right)|X_{n}\right\rangle$$

and

(31) $${\tilde{R}}_{Y}\left(r|X_{n}\right)={\left\langle {\tilde{Y}}^{\prime }\left(x\right){\tilde{Y}}^{\prime }\left(x+r\right)|X_{n}\right\rangle }_{r=\left|r\right|}$$

## 3. Results

**Figure 1:**
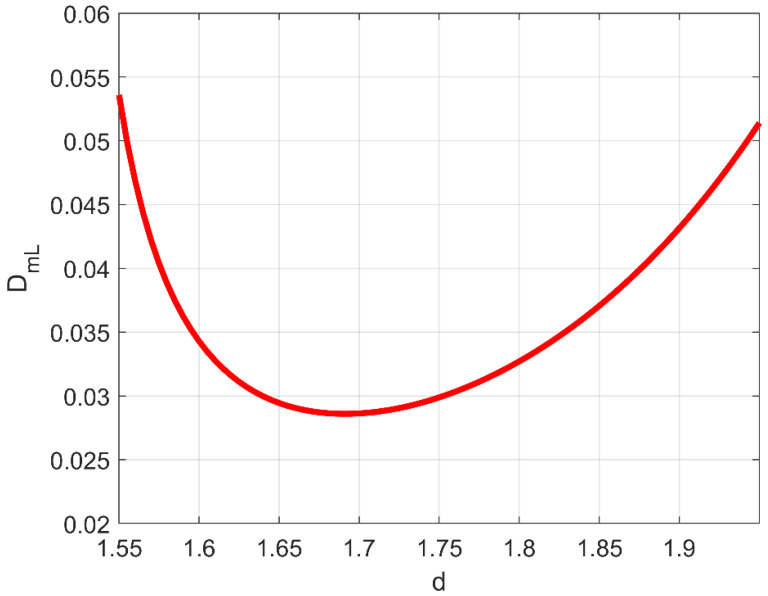
Normalized longitudinal macrodispersion coefficient in evolving-scale, anti-persistently correlated (0 < *b* < 1) log-conductivity distributions as a function of the corresponding (single) fractal dimension (Equation (3), from [3]).

**Figure 2:**
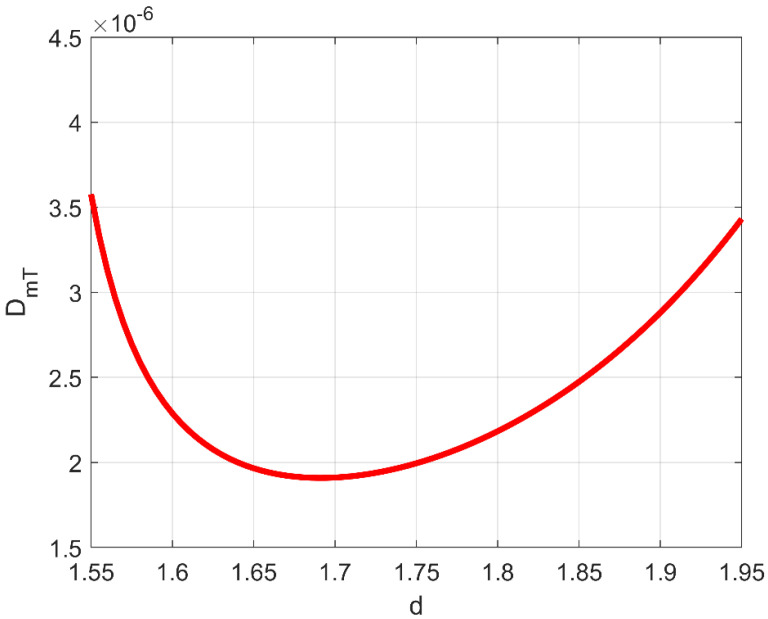
Normalized transverse macrodispersion coefficient in evolving-scale, anti-persistently correlated (0 < *b* < 1) log-conductivity distributions as a function of the corresponding (single) fractal dimension (Equation (4), from [3]).

Figure 1 and Figure 2 respectively show D_mL_ (3) and D_mT_ (4).

As one can clearly see from these figures, both D_mL_ and D_mT_ exhibit a minimum exactly at *d* ≅ 1.7, meaning that, in the so-called anti-persistent range (0<b<1, 1.5<d<2), macrodispersion intensity in fractal porous formations does not vary monotonically as a function of their self-similar geometrical complexity, and (more importantly) that the clinically detected diffusion-limited fractal dimension in late-stage cancerous lesions (which is also reminiscent of the colloidal state of solutions (e.g., [1])) likely constitutes a universal nature imprint. In Figure 1 and Figure 2, and based on the typical values of mean velocity *U*, pure diffusion coefficient *D*_0_, and representative dimension of the related fractal domain *L* = 1/*k*_0_, the Péclet number was assumed equal to 10^4^. Note that, with the functional dependence on *d* and *Pe* in Equations (3) and (4) being completely decoupled, a smaller or a larger Péclet number would in no way compromise the existence of the relative minimum at *d* ≅ 1.7. Additionally, as can be inferred from the following simple example, the dominant longitudinal macrodispersion coefficient is practically independent of a ubiquitous, reasonably large Péclet. In an aqueous solution, typical pure-diffusion coefficients are in the range of 10^−10^ to 10^−9^ m^2^/s. In [3], the well-known 1985–1988 Cape Cod, Massachusetts, solute transport experiment was revisited in terms of a fractal sand/gravel log-conductivity distribution, with a physical upper cutoff represented by the depth of the sedimentary layer where plume transport took place (30 m). The structural anisotropy ratio (vertical to horizontal single-scale correlation length) was equal to 0.19; the average longitudinal velocity *U* was equal to about 2.8 × 10^−6^ m/s. The corresponding Peclét number would be, in this case, $Pe=2.8\cdot 10^{-6}30/\left(0.19\cdot 10^{-10}\right)≅4.4\cdot 10^{6}$, with $\left(0.5-20/3Pe\right)=0.499998$ in Equation (3). It has to be emphasized that even a *Pe* precautionarily assumed to be equal to the order of 10^4^ would lead to $\left(0.5-20/3Pe\right)=0.499333$.

From a phenomenological point of view, one may infer that solute spreading in geologic formations undergoes a gradual transition in terms of driving mechanisms, spanning from highly channelized flow and transport in fractured carbonate rocks (smaller fractal dimensions) to Darcian flow and transport in evolving-scale cohesionless deposits (larger fractal dimensions). The minimum would manifest itself in correspondence with the aggregation-state transition when, while the magnitude of the channel-like dispersion is decreased due to the reduction in fracture width, the number, the tortuosity, and the degree of connectivity of the micro-channels is still not sufficient to trigger a truly two-phase medium dispersion.

Similarly, as already argued by [1], the advanced stages of cancerous tissue evolution seem to intriguingly represent a sort of intermediate condition between two ideal extremes: the suspension (with the cells that would practically be dispersed in the extracellular matrix and fed by a large mesh-size vascular network characterized by reduced branching and tortuosity) and the gel (with the cells that would be very densely aggregated and fed by a small mesh-size vascular network characterized by pronounced branching and tortuosity). Following the proposed hydrogeologic analogy, one might conclude that, in the first ideal limiting case, oxygen and nutrient dispersion would almost exclusively be an intravascular process; conversely, in the second ideal limiting case, it would almost exclusively be an extravascular process, with frequent two-way capillary/cell exchanges and very poor tissue oxygenation. As a consequence, the diffusion-limited fractal dimension characterizing the advanced stages of cancerous lesion progression may be representative of a tissue architecture characterized by the maximum cell aggregation that still allows for the (minimum) vital metabolic supply.

### 3.1. Comparison with the Outcome of Clinical Surveys

**Figure 3:**
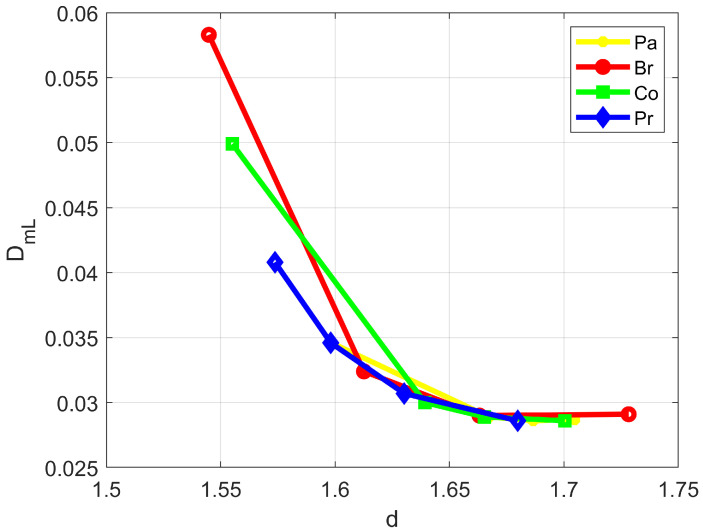
Normalized longitudinal macrodispersion coefficient in some cancerous tissues at different progression stages as a function of the corresponding average fractal dimensions reported by [2] (Pa: pancreas; Br: breast; Co: colon; Pr: prostate).

Figure 3 shows (with the mean velocity *U* and fractality scale 1/*k*_0_ being the same) the evolution of the Fickian D_mL_ (Equation (3)) as a function of the average fractal dimensions reported by [2] and referring to four different types of cancerous tissue (pancreas, breast, colon and prostate) at four different progression stages, including the healthy (pre-cancerous) condition.

As the figure highlights, a drastic reduction in the longitudinal dispersion coefficient would take place between the pre-cancerous and the first cancerous stage, with a subsequent residual decrease toward almost constant values. Table 1 and Table 2 numerically quantify the global percentage reduction $∆D_{mL}=\left(D_{mL4}-D_{mL1}\right)/D_{mL1}$ of the longitudinal and the transverse dispersion coefficient as calculated from Equations (3) and (4).

The values listed in the third column of the two tables are compared here, where available, with the outcome of imaging-based clinical surveys. Results from contrast ultrasound dispersion imaging (CUDI) for the assessment of the perfusion-related dispersion parameter *K* = *U*^2^/*D* in prostate cancer (the symbols are here reported in their original version) were proposed by [16]. The detected dispersion values *D*, which were obtained by adopting a locally one-dimensional transport model, were in line with the dispersion parameter *K* increasing in the presence of cancer. Specifically, the authors reported *K* = 0.37 ± 0.08 s^−1^ for benign and *K* = 1.01 ± 0.77 s^−1^ for malignant tissue, allowing (in the case of almost constant advective velocity *U*) for an estimation of an average 63.3% reduction in *D* due to the pathologic condition. Table 1 and Table 2 in the present study, in which the mean velocity is evaluated at the tissue scale, respectively yield, for prostate, the more conservative −29.9% and −29.83%, respectively. The overall decrease in the perfusion-related dispersion coefficient estimated by diffusion weighted imaging (DWI) from a low to high Ki-67 marker (a routinely employed global indicator of cancerous lesion progression) can also be assessed from Table 2 in [17]. The reported lung cancer values were $D^{*}=0.0231\pm 0.0127\;{\mathrm{mm}}^{2}/s$ at low Ki-67 and $D^{*}=0.0167\pm 0.00807\;{\mathrm{mm}}^{2}/s$ at high Ki-67, leading to an average percentage reduction $∆D^{*}\%=-27.7$. No specific comparison could be performed in this case, since lung cancer was not covered by the pioneer fractal dimension investigation [2]. Nevertheless, a 27.7% $D^{*}$ reduction seems to be reasonably in line with the values listed in Table 1 and Table 2 of the present study, which provide average values of around −35%. A similar estimation can be made for pancreatic cancer from Table 3 in [18]. The reported DWI perfusion-related dispersion coefficients in this case were $D^{*}=0.001356\pm 0.000573\;{\mathrm{mm}}^{2}/s$ in normal pancreatic parenchyma and $D^{*}=0.001128\pm 0.000566\;{\mathrm{mm}}^{2}/s$ in pancreatic tumor, with an average $∆D^{*}$% = −16.8. Note, in this third case, the very good agreement between the reported $D^{*}$ average percentage reduction and the percentage reduction given in Table 1 (−17.05%) and Table 2 (−17.06%) of the present study for longitudinal and transverse macrodispersion coefficient in the presence of pancreatic cancer. The reason for the definitely better agreement between clinical observations and theoretical predictions in the case of DWI measurements (pancreatic cancer results presented by [18]) compared to the CUDI counterpart (prostate cancer results presented by [16]) may precisely be due to the different methodology. As a matter of fact, and as mentioned above, measurements from CUDI (which is an intravascular contrast-agent dynamic investigation) are interpreted based on a 1-D, conduit-like transport model. DWI dispersion measurements are obtained after a filtering operation that separates water apparent diffusion coefficient ADC into a perfusion-related pseudo-diffusion coefficient $D^{*}$ and a true-diffusion coefficient D. The mathematical operative relationship (see [18]), here reported in the original notation, is:

(32) $$S\left(b\right)=S_{0}\exp\left(-b\mathrm{ADC}\right)=S_{0}\left[\left(1-f\right)\exp\left(-bD\right)+f\exp\left(-bD^{*}\right)\right]$$

where S is the magnetic resonance signal at the given radio-frequency *b*, $S_{0}$ the signal in the absence of radio-frequency saturation, and *f* the pseudo-diffusion fraction. In other words, DWI methodology treats the tissue like an equivalent continuous medium made of micro-vessels (fraction *f*, which contributes with $D^{*}$) and cells/extracellular matrix (fraction 1 − *f*, which contributes with *D*). This is clearly more similar to what the geostatistical approach does, with medium porosity *η* that plays the role of *f*. The only difference is that the aquifer solid fraction 1 − *η* is totally impervious and is not subject to any kind of diffusion process.

Overall, the uncalibrated porous media fluid-dynamic model seems to be able to properly grasp the order of magnitude of the percentage reduction in the tissue perfusion-related dispersion coefficient due to cancerous conditions, and to reproduce the unambiguously detected negative trend that characterizes its relationship with disease progression. This negative trend is confirmed by almost all the documented experimental investigations, where it is concordantly explained by an increase in microvascular tortuosity, and the consequent limitation of the dispersion kinetics represented by the dispersion coefficient. A few exceptions are reported in the literature for specific types of tumors, like those in the brain (e.g., [19]).

### 3.2. Perspective Utilization—Multifractal Extension of the Macrodispersion Theory

As explained by [16], the local (voxel-scale) evaluation of the perfusion-related dispersion coefficient may allow for the detailed mapping of tissue architecture. A multi-fractal extension of the above-discussed fluid-dynamic dispersion theory may therefore help decode the resulting maps by mathematically relating the local values of the macrodispersion coefficients to the local (voxel-scale) tissue/micro-vessel network fractal dimension. The key transformation consists in switching from a continuous hierarchy of stationary log-conductivity fields to a non-stationary one. Note that, in geostatistics, the term “non-stationary” refers to random variable correlation functions that depend on both the distance between the correlated points and the exact position of one of them. The suitable generalization of Equation (6) then reads:

(33) $${\tilde{\gamma }}_{Y}\left(r,X\right)=a\left(X\right)r^{b\left(X\right)}=\int_{0}^{\infty }\frac{\partial }{\partial \lambda }\left[\gamma_{Y}\left(r,\lambda ,X\right)\right]d\lambda =\int_{0}^{\infty }\frac{\phi \left(X\right)}{\lambda^{1+b\left(X\right)}}\left[1-\exp\left(-r\lambda \right)\right]d\lambda$$

where $b\left(X\right)$ indicates the space-dependent scaling exponent, and the single-scale variance and covariance are now respectively given by:

(34) $$d\sigma_{Y}^{2}\left(X,\lambda \right)=\frac{\phi \left(X\right)}{\lambda^{1+b\left(X\right)}}d\lambda ;dR_{Y}\left(r,X,\lambda \right)=\frac{\phi \left(X\right)}{\lambda^{1+b\left(X\right)}}\exp\left(-r\lambda \right)d\lambda$$

It is considered that:

(35) $$\phi \left(X\right)=-\frac{a\left(X\right)}{\Gamma \left[-b\left(X\right)\right]}=k_{0}^{b\left(X\right)}$$

with *k*_0_ here indicating the wave-length cutoff related to the cell cluster representative dimension $1/k_{0}$.

In view of the detection grid discretization, the absolute coordinate X is assumed to be given by the sum of a local (voxel-scale) coordinate **x** and a global coordinate nl identifying the center of each voxel: X=x+nl. The single-scale random log-conductivity $Y\left(X,\lambda \right)=\langle Y\rangle +Y^{\prime }\left(X,\lambda \right)$ is considered to be stationary in **x** and to be affected by a slow deterministic trend in **n**. In other words, the single log-conductivity hierarchical component is considered to be approximately stationary at the detection-cell scale, with:

(36) $$dR_{Y}\left(r,\lambda |X_{n}\right)=\frac{\phi \left(X_{n}\right)}{\lambda^{1+b\left(X_{n}\right)}}\exp\left(-r\lambda \right)d\lambda$$

In these conditions, and by virtue of the superposition principle, each detection cell turns out to be characterized by a practically constant scaling exponent and associated fractal dimension. The plausible condition $1/k_{0}\ll l$ (note that $1/k_{0}$ is a characteristic cellular scale, whereas *l* is a by-eye visible length) then enables the locally (intra-detection cell) asymptotic transport approach. The resulting nonergodic macrodispersion coefficients are obtained as the local (voxel-scale) declination of Equation (3) and Equation (4) based on the locally stationary generalization expressed by Equations (27)–(31):

(37) $$D_{mL}\left(X_{n}\right)=\frac{D_{mL}\left(X_{n}\right)k_{0}}{U}=\frac{B\left(\frac{7-2d\left(X_{n}\right)}{2},\frac{2d\left(X_{n}\right)-3}{2}\right)}{\left(6-2d\left(X_{n}\right)\right)4^{\left(5-2d\left(X_{n}\right)\right)/2}\pi^{5-2d\left(X_{n}\right)}}\left(\frac{1}{2}-\frac{20}{3Pe}\right)$$

(38) $$D_{mT}\left(X_{n}\right)=\frac{D_{mT}\left(X_{n}\right)k_{0}}{U}=\frac{B\left(\frac{7-2d\left(X_{n}\right)}{2},\frac{2d\left(X_{n}\right)-3}{2}\right)}{3\left(6-2d\left(X_{n}\right)\right)4^{\left(5-2d\left(X_{n}\right)\right)/2}\pi^{5-2d\left(X_{n}\right)}Pe}$$

Equations (37) and (38) practically interpret the macroscopic dispersion coefficients in multifractal log-conductivity distributions as sort of constitutive variables that synthesize, at grid scale, the effects of the sub-grid fractal heterogeneity.

Once the experimental dispersion coefficient map is obtained, it has to be normalized by taking its ratio to the healthy counterpart $D_{H}$ (note that this operation is needed in order to make the map independent of the scale $k_{0}/U$, which would likely be of rather difficult experimental determination):

(39) $$\frac{\left(D_{1},D_{2},\ldots ,D_{M}\right)}{D_{H}}=\left(D_{1},D_{2},\ldots ,D_{M}\right)$$

where *M* indicates the total number of detection cells. The experimental assessment of the longitudinal dispersion coefficients may be pursued by evaluating, at a time large enough to allow for the complete tissue saturation, the variation in the longitudinal central inertia moment $I_{Ln}$ (during a properly small time-discretization interval ∆t) of the labelled contrast agent particles that happen to lie within each detection cell:

(40) $$I_{Ln}\left(t+∆t\right)=I_{Ln}\left(t\right)+2D_{n}∆t=\sum_{p=1}^{N_{n}}\frac{{\left[X_{1p}\left(t+∆t\right)-{\bar{X}}_{1}\left(t+∆t\right)\right]}^{2}}{N}$$

In Equation (40), $X_{1p}$ indicates the longitudinal coordinate of the generic particle, $N_{n}$ is their total number, and the barycentre ${\bar{X}}_{1}$ is given by:

(41) $${\bar{X}}_{1}\left(t+∆t\right)=\sum_{p=1}^{N}\frac{X_{1p}\left(t+∆t\right)}{N}$$

**Figure 4:**
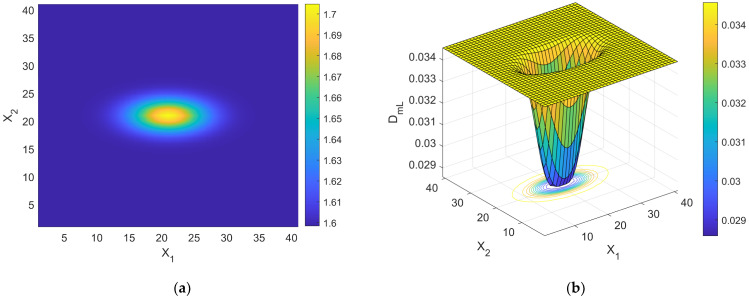
(**a**) Shading-interpolated map of the hypothetical Gaussian-shaped anomalous distribution of fractal dimensions in a partially cancerous tissue, built by combining the data referring to the 4 different progression stages reported by [2] for pancreas; the detection grid spacing *l* is used as the spatial scale; ${∆}_{1}=4l,\;{∆}_{2}={∆}_{3}=2l$. (**b**) Surface-plot of the distribution of normalized macrodispersion coefficients corresponding to the distribution of fractal dimensions shown in Figure 4a; the detection grid spacing *l* is used as the spatial scale.

**Figure 5:**
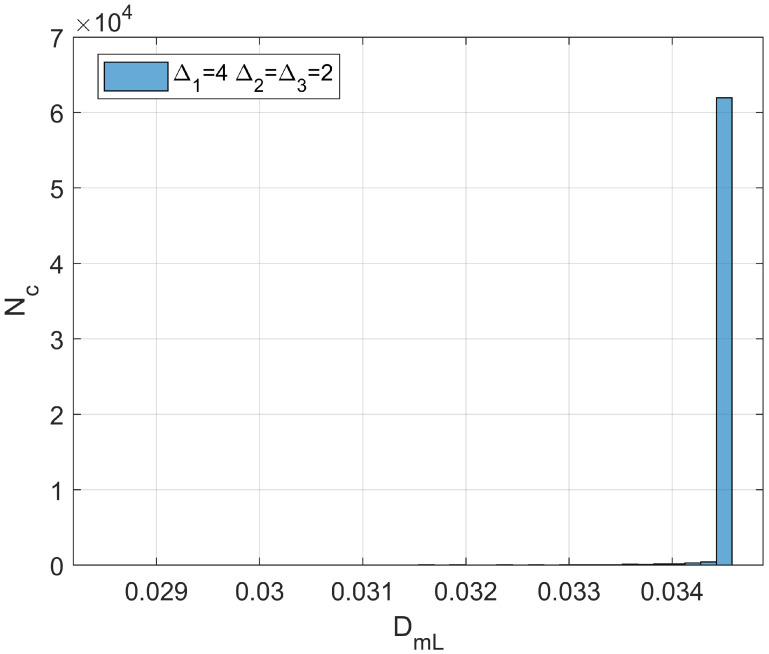
Frequency histogram of function $D_{mL}\left(X_{n}\right)$ as a function of the number of cells (N_c_) characterized by the different classes of its values within the selected 40*l* × 40*l* × 40*l* detection grid. ${∆}_{1}=4l,{∆}_{2}={∆}_{3}=2l$.

**Figure 6:**
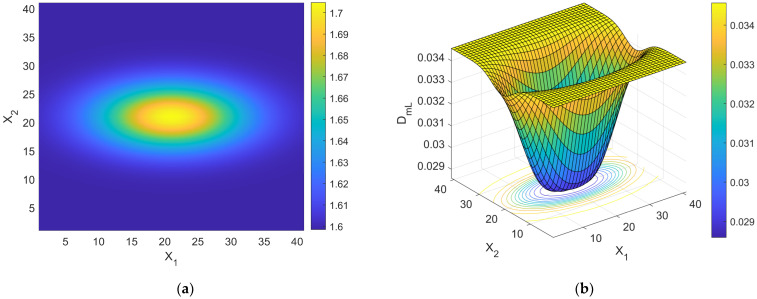
(**a**) Shading-interpolated map of the hypothetical Gaussian-shaped anomalous distribution of fractal dimensions in a partially cancerous tissue, built by combining the data referring to the 4 different progression stages reported by [2] for pancreas; the detection grid spacing *l* is used as the spatial scale; ${∆}_{1}=8l,\;{∆}_{2}={∆}_{3}=4.$ (**b**) Surface-plot of the distribution of normalized macrodispersion coefficients corresponding to the distribution of fractal dimensions shown in Figure 6a; the detection grid spacing *l* is used as the spatial scale.

**Figure 7:**
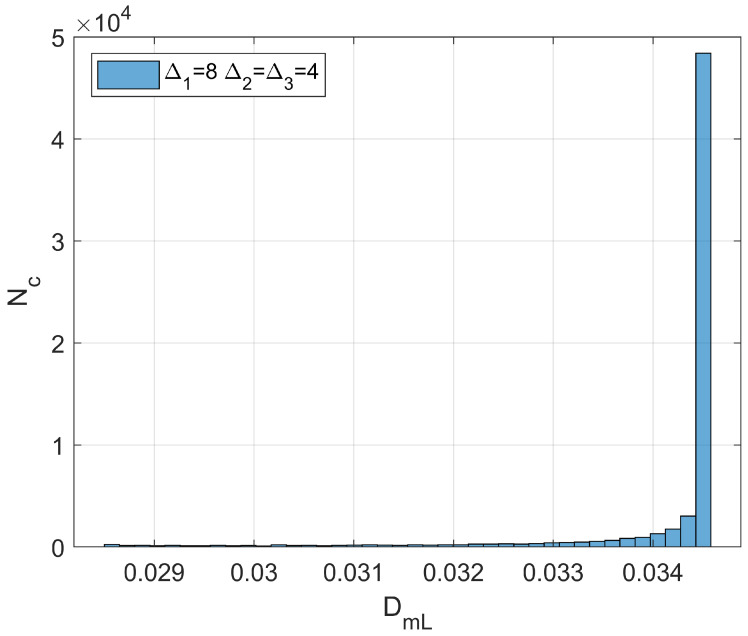
Frequency histogram of function $D_{mL}\left(X_{n}\right)$ as a function of the number of cells (N_c_) characterized by the different classes of its values within the selected 40*l* × 40*l* × 40*l* detection grid. ${∆}_{1}=8l,\;{∆}_{2}={∆}_{3}=4l$.

**Figure 8:**
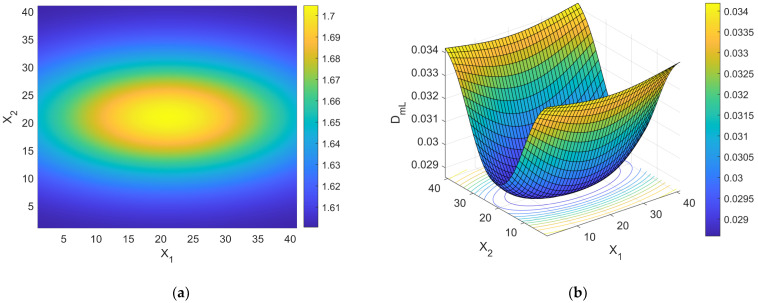
(**a**) Shading-interpolated map of the hypothetical Gaussian-shaped anomalous distribution of fractal dimensions in a partially cancerous tissue, built by combining the data referring to the 4 different progression stages reported by [2] for pancreas; the detection grid spacing *l* is used as the spatial scale; ${∆}_{1}=16l,\;{∆}_{2}={∆}_{3}=8l$. (**b**) Surface-plot of the distribution of normalized macrodispersion coefficients corresponding to the distribution of fractal dimensions shown in Figure 8a; the detection grid spacing *l* is used as the spatial scale.

**Figure 9:**
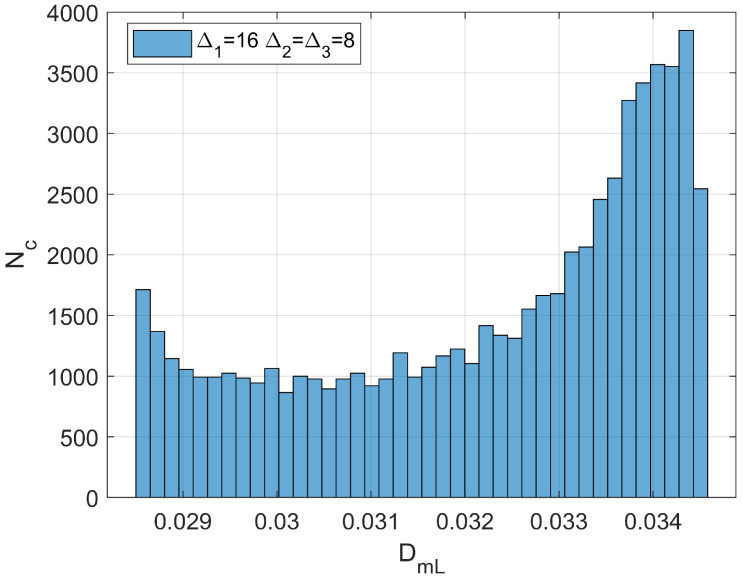
Frequency histogram of function $D_{mL}\left(X_{n}\right)$ as a function of the number of cells (N_c_) characterized by the different classes of its values within the selected 40*l* × 40*l* × 40*l* detection grid. ${∆}_{1}=16l,\;{∆}_{2}={∆}_{3}=8l$.

Since $D_{mL}$ is much larger than $D_{mT}$, it can therefore be considered exhaustively representative of solute spreading dynamics. Then, from Equation (39), with:

(42) $$\left(D_{1},D_{2},\ldots ,D_{M}\right)=\frac{D_{mL}\left[d\left(X_{n}\right)\right]}{D_{mL}\left(d_{H}\right)}$$

one should be able to reconstruct the spatial distribution of *d* by simple algebraic algorithms. It is worth noting that the branch of the curve $D_{mL}=D_{mL}\left(d\right)$ in Figure 1 to be used for the estimation procedure is the descending one, provided that cancer lesion progression is a fractal dimension-increasing process, with an upper threshold associated with the diffusion-limited condition. As an illustrative example referring to pancreatic cancer stages as reported by [2], a hypothetical Gaussian-shaped distribution of fractal dimensions was built by:

(43) $$d\left(X\right)=d_{1}+\left(d_{4}-d_{1}\right)\prod_{i=1}^{3}\frac{1}{\sqrt{2\pi {∆}_{i}^{2}}}\exp\left[-\frac{{\left(X_{i}-X_{ci}\right)}^{2}}{2{∆}_{i}^{2}}\right]$$

where $X_{ci}$ indicates the *i*th component of the neoplasm center and ${∆}_{i}^{2}$ the associated inertia moment. In the reported case, $d_{1}=1.5984$ (pre-cancerous average fractal dimension) and $d_{4}=1.7047$ (fourth-stage average fractal dimension). The selected anomaly center coordinates were $X_{c1}=X_{c2}=X_{c3}=20l$. Figure 4a,b and Figure 5 respectively show the maps of the (X_1_, X_2_) slice of this distribution, the surface-plot of the associated longitudinal macrodispersion coefficients, and the corresponding frequency histogram of $D_{mL}\left(X_{n}\right)$ as a function of the number of cells characterized by the different classes of its values within the selected 40*l* × 40*l* × 40*l* detection grid, for ${∆}_{1}=4l,\;{∆}_{2}={∆}_{3}=2l$. Figure 6a,b and Figure 7 respectively show the maps of the (X_1_, X_2_) slice of this distribution, the surface-plot of the associated longitudinal macrodispersion coefficients, and the corresponding frequency histogram of $D_{mL}\left(X_{n}\right)$ as a function of the number of cells characterized by the different classes of its values within the selected 40*l* × 40*l* × 40*l* detection grid, for ${∆}_{1}=8l,\;{\;∆}_{2}={∆}_{3}=4l$. Finally, Figure 8a,b and Figure 9 respectively show the maps of the (X_1_, X_2_) slice of this distribution, the surface-plot of the associated longitudinal macrodispersion coefficients, and the corresponding frequency histogram of $D_{mL}\left(X_{n}\right)$ as a function of the number of cells characterized by the different classes of its values within the selected 40*l* × 40*l* × 40*l* detection grid, for ${∆}_{1}=16l,\;{\;∆}_{2}={∆}_{3}=8l$. Note that the choice of the pancreas progression sequence was suggested by the above-discussed almost perfect agreement between the detected and predicted average percentage reduction in the dispersion coefficients.

As one can see from comparison between the fractal dimension maps and the macrodispersion coefficient surface-plots, this sort of fluid-dynamic “transfer function” has the advantage of emphasizing the anomaly fringe gradient (the surface-plot sinking is clearly more extended than the map’s lit area, particularly for the smaller ${∆}_{i}$) while returning a flatter central core. Therefore, it proves to be potentially more efficient in assessing anomaly effective contours, especially at early progression stages. The $D_{mL}\left(X_{n}\right)$ histograms display initially almost one side Dirac-like distributions centered around the maximum (healthy) $D_{mL}$ value. As the dimensions of the anomaly increase, a marked bimodality tends to manifest itself, with the gradual increase of a second peak at the lower extreme of the $D_{mL}$ range and a simultaneous though slower gain in the intermediate classes. Note that the simple calculation of the percentage of detection cells sampling the different $D_{mL}$ values may straightforwardly be related to the percentage of the monitored tissue characterized by different values of the fractal dimension (and, therefore, by different cancerous lesion progression stages).

**Figure 10:**
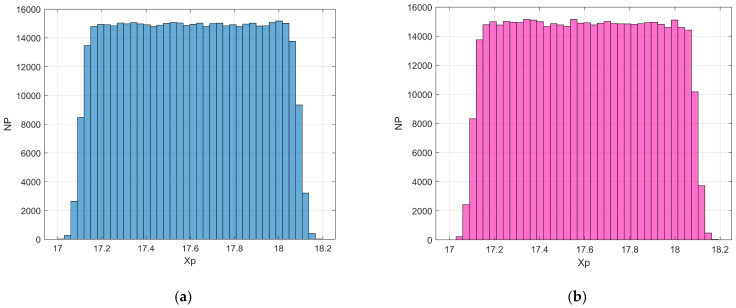
(**a**) Frequency histogram of particles’ longitudinal position obtained from 3-D particle tracking that started from a randomly uniform distribution in the detection cell centered on $X_{1}=X_{2}=X_{3}=17.5l$; $∆t=0.1l/U,\;{∆}_{1}=4l,\;{∆}_{2}={∆}_{3}=2l$. (**b**) Frequency histogram of particles’ longitudinal position obtained from 3-D particle tracking that started from a randomly uniform distribution in the detection cell centered on $X_{1}=X_{2}=X_{3}=17.5l$; $∆t=0.1l/U,\;{∆}_{1}=16l,\;{∆}_{2}={∆}_{3}=8l$.

**Figure 11:**
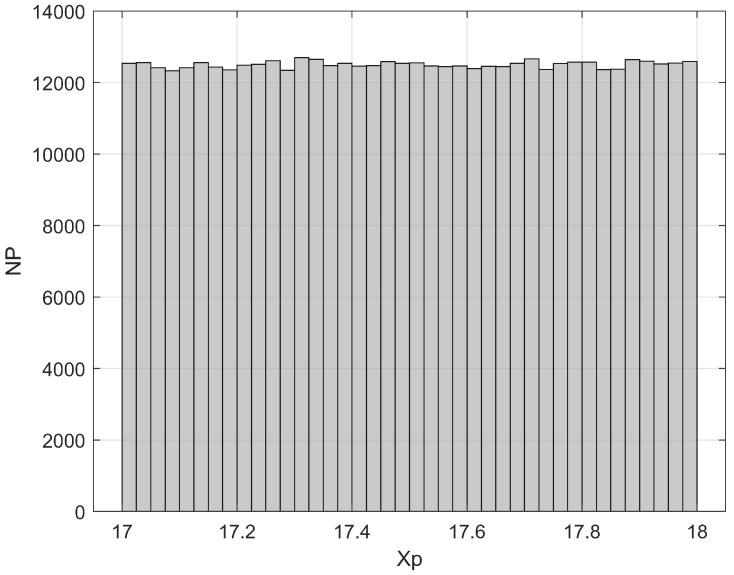
Frequency histogram of particles’ initial position corresponding to a randomly uniform distribution in the detection cell centered on $X_{1}=X_{2}=X_{3}=17.5l$.

Finally, as an example of a detection cell-scale fractal dimension estimate based on the suggested procedure, Figure 10a,b respectively show (for ${∆}_{1}=4l,\;{\;∆}_{2}={∆}_{3}=2l$ and ${∆}_{1}=16l,\;{\;∆}_{2}={∆}_{3}=8l$) the frequency histogram of particles’ longitudinal position obtained from a 3-D particle tracking that started from a randomly uniform distribution (simulating a local equilibrium condition) in the detection cell centered on $X_{1}=X_{2}=X_{3}=17.5l$ after the time interval ∆t=0.1l/U. It was assumed that $l=10/k_{0}$ and, therefore, $∆t=1/k_{0}U.$ For the sake of comparison, Figure 11 shows the frequency histogram of the initial positions.

The number of particles used to reproduce the movement of the tracer spot was *N* = 500,000. This high number was justified by the need to stabilize the results. An alternative approach would have been considering a lower number of particles that started at the given cell, and then computing the longitudinal macrodispersion coefficient as the ensemble average over multiple realizations of the experiment. The numerical algorithm that simulated the movement of particles due to a unit velocity U, a unit grid spacing *l*, and the space-varying $D_{mL}\left(X\right)=D_{mL}\left(X\right)/\left(lk_{0}\right)$, $D_{mT}\left(X\right)=D_{mT}\left(X\right)/\left(lk_{0}\right)$ was:

(44) $$X_{1p}\left(t+∆t\right)=X_{1p}\left(t\right)+U∆t+\sqrt{2D_{mL}\left[X_{1p}\left(t\right),X_{2p}\left(t\right),X_{3p}\left(t\right)\right]∆t}N\left(0,1\right)$$

(45) $$X_{2p}\left(t+∆t\right)=X_{2p}\left(t\right)+\sqrt{2D_{mT}\left[X_{1p}\left(t\right),X_{2p}\left(t\right),X_{3p}\left(t\right)\right]∆t}N\left(0,1\right)$$

(46) $$X_{3p}\left(t+∆t\right)=X_{3p}\left(t\right)+\sqrt{2D_{mT}\left[X_{1p}\left(t\right),X_{2p}\left(t\right),X_{3p}\left(t\right)\right]∆t}N\left(0,1\right)\;$$

where $N\left(0,1\right)$ indicates the generic element of a standard normal distribution. The above Brownian non-Gaussian random walk scheme should be viewed as an extension of the truly Gaussian case, with trajectory-fluctuating dispersion coefficients and locally symmetric steps in space. See [20,21] for an exhaustive discussion about Brownian non-Gaussian diffusion in heterogeneous media. Based on Equations (40) and (41), the following was obtained:

(47) $$\frac{D_{mLn}}{D_{mL}\left(d_{H}\right)}=\frac{0.0032}{0.0034}=0.94$$

for ${∆}_{1}=4l,\;{∆}_{2}={∆}_{3}=2l$ (Figure 4a,b and Figure 5) and

(48) $$\frac{D_{mLn}}{D_{mL}\left(d_{H}\right)}=\frac{0.0029}{0.0034}=0.853$$

for ${∆}_{1}=16l,\;{∆}_{2}={∆}_{3}=8l$ (Figure 8a,b and Figure 9). From Equation (3), the reduction in the healthy longitudinal macrodispersion coefficient (fractal dimension d=1.5984) of 6% and 14.7%, respectively, resulted in an estimated altered fractal dimension d=1.62 in the first case and d=1.69 in the second, with the detection cell being the same.

## 4. Discussion

The present study aimed at suggesting the existence of a universal diffusion/dispersion-limited aggregation-state of two-phase systems that may authorize the adoption of the mathematical expression of the normalized nonergodic macrodispersion coefficients (which were previously derived by the author for upper-bounded fractal log-conductivity distributions in saturated porous media) as physically-based descriptors of blood perfusion dynamics in cancerous tissues.

The author’s recent stochastic investigation indeed demonstrated that tracer dispersion in evolving-scale, anti-persistently correlated log-conductivity distributions is non-monotonically related to the corresponding fractal dimension *d*. Instead, it showed that the Fickian (and, therefore, asymptotically constant) component of both the effective longitudinal and the transverse macrodispersion coefficient exhibit a clearly detectable minimum at d≅1.7. Based on recent clinical surveys, the same critical fractal dimension (besides being notoriously representative of the colloidal state of solutions) seems to characterize the late stages of cellular arrangement in cancerous tissues.

One may therefore conclude that both geological formations and evolving biological tissues undergo a gradual transition from one perfusion-related dispersion driving mechanism to the other. As a matter of fact, geological formations range from fractured carbonate rocks, where dispersion essentially takes place within large subsurface channels that are spaced well apart, to cohesionless sedimentary deposits where dispersion is a truly two-phase process. Similarly, biological tissues may ideally be thought of as ranging from weakly aggregated populations of cells fed by large mesh-size vessel networks within the extracellular matrix to densely and pathologically aggregated populations of cells fed by small mesh-size vessel networks. In the first case, perfusion would mainly be intravascularly controlled; in the second case, perfusion would mainly be extra-vascularly controlled by anoxia-prone multiple capillary/cell exchanges. The diffusion-limited fractal dimension characterizing the advanced stages of cancerous lesions might therefore be interpreted as the expression of a tissue architecture characterized by the maximum cell aggregation that still allows for the minimum vital metabolic supply.

In natural flow fields there exist at least two striking examples of dynamic system micro-structural adjustments targeted to the optimization of the underlying driving mechanism: (1) according to Kolmogorov’s equilibrium theory (e.g., [22]), the adaptation of the turbulent micro-scales to the energy transmitted by the largest scales of vorticity to guarantee a commensurate dissipation, and (2) the tendency of rivers to modify local slope and morphology in such a way as to achieve an equilibrium condition (e.g., [23]) in which neither erosion nor deposition takes place.

Although the current stochastic analytical framework and the current clinical survey set-up are characterized by somehow different underlying conceptual models, a quantitative comparison in terms of perfusion-related dispersion coefficients between theoretical predictions and the outcome of CUDI and DWI measurements was attempted. Besides the full agreement about the negative average correlation between perfusion-related dispersion intensity and cancer progression (in the present study, expressed by the increasing tissue fractal dimension), the comparison revealed the overall good performance of the theory also in capturing the order of magnitude of the corresponding average percentage reduction.

Going into detail, magnetic resonance (DWI) measurements seem to be definitely more in line with theoretical model predictions. The reason has to be searched for in the specificity of this methodology, which interprets the biological tissue as an equivalent continuum characterized by a pseudo-diffusive fraction (micro-circulation domain) and a truly diffusive fraction (cell/extracellular matrix domain). The approach of the continuum equivalent precisely constitutes the landmark assumption of the geostatistical hydrogeology and the associated macro-dispersion theory.

Finally, a multi-fractal extension of this theory was proposed to encompass its possible perspective utilization as an interpretative diagnostic tool. Interestingly enough, the tissue map obtained from the space-dependent version of the nonergodic longitudinal macrodispersion coefficient for a hypothetical Gaussian-shaped neoplasm (and, therefore, a Gaussian-shaped fractal dimension anomaly) suggests that this important fluid-dynamic “transfer function” has the advantage of emphasizing the tissue anomaly fringe gradient, thus proving to be particularly efficient in more clearly assessing neoplasm contours, especially at early progression stages.

To the author’s knowledge, systematic clinical point estimates of the perfusion-related dispersion coefficient in biological tissues are still not available. Therefore, the most straightforward and logical expansion of the present investigation would consist of its validation against the outcome of detailed experimental surveys, possibly performed according to the suggested methodology in the presence of heterogeneous fractal dimension distributions. The results of this validation could also inspire suitable model calibration, improvements, and modifications.

## Figures and Tables

**Table 1:** Percentage reduction in the normalized longitudinal macrodispersion coefficient for the different types of cancer shown in Figure 3.

Type of Cancer	$D_{mL1}$	${∆D}_{mL}\%$
Pancreas	0.0346	−17.05
Breast	0.0583	−50.08
Colon	0.0499	−42.69
Prostate	0.0408	−29.9
**Average**	**0.0459**	−**34.93**

**Table 2:** Percentage reduction in the normalized transverse macrodispersion coefficient for the different types of cancer whose longitudinal counterparts are shown in Figure 3.

Type of Cancer	$D_{mL1}$	${∆D}_{mL}\%$
Pancreas	0.0023074	−17.06
Breast	0.0038929	−50.05
Colon	0.0033278	−42.57
Prostate	0.0027255	−29.83
**Average**	**0.0030634**	−**34.99**

## Data Availability

All the correlated data and the utilized mathematical tools are made available in the article.

## References

[1] Leggett S.E., Neronha Z.J., Bhaskar D., Sim J.Y., Myrto Perdikari T., Wong I.Y. (2019). Motility-limited aggregation of mammary epithelial cells into fractal-like clusters. Proc. Natl. Sci. Acad. USA.

[2] Elkington L., Adhikari P., Pradhan P. (2022). Fractal dimension analysis to detect the progress of cancer using transmission optical microscopy. Biophysica.

[3] Pannone M. (2023). Theoretical investigation of nonergodic solute dispersion in natural porous formations characterized by persistent and antipersistent power-law log-conductivity correlations. Hydrogeol. J..

[4] Pannone M., Kitanidis P.K. (1999). Large-time behavior of concentration variance and dilution in heterogeneous formations. Water Resour. Res..

[5] Pannone M., Kitanidis P.K. (2004). On the asymptotic behavior of dilution parameters for Gaussian and hole-Gaussian log-conductivity covariance functions. Transp. Porous Media.

[6] Ditlevsen O. (1981). Uncertainty Modelling with Applications to Multidimensional Civil Engineering Systems.

[7] Khaled A.R.A., Vafai K. (2003). The role of porous media in modeling flow and heat transfer in biological tissues. Int. J. Heat Mass Transf..

[8] Pannone M. (2021). Modeling Left Ventricle Perfusion in Healthy and Stenotic Conditions. Bioengineering.

[9] Feder J. (1988). Fractals.

[10] Painter S., Mahinthakumar G. (1999). Prediction uncertainty for tracer migration in random heterogeneities with multifractal character. Adv. Water Res..

[11] Gradshteyn I.S., Ryzhik I.M. (1994). Table of Integrals, Series, and Products.

[12] Walters P. (1982). An Introduction of Ergodic Theory.

[13] Pannone M. (2020). A theoretical study about ergodicity issues in predicting contaminant plume evolution in aquifers. Water.

[14] Dagan G. (1989). Flow and Transport in Porous Formations.

[15] Rubin Y. (2003). Applied Stochastic Hydrogeology.

[16] Mischi M., Vijkstra H. Contrast dispersion imaging for cancer localization. Proceedings of the 2014 36th Annual International Conference of the IEEE Engineering in Medicine and Biology Society.

[17] Zheng Y., Huang W., Zhang X., Lu C., Fu C., Li S., Liu G. (2021). A noninvasive assessment of tumour proliferation in lung cancer patients using intravoxel incoherent motion. Magn. Reson. Imaging J. Cancer.

[18] Granata V., Fusco R., Sansone M., Grassi R., Maio F., Palaia R., Tatangelo F., Botti G., Grimm R., Curley S. (2020). Magnetic resonance imaging in the assessment of pancreatic cancer with quantitative parameter extraction by means of dynamic contrast-enhanced magnetic resonance imaging, diffusion kurtosis imaging and intravoxel incoherent motion diffusion-weighted imaging. Ther. Adv. Gastroenterol..

[19] Ima M. (2021). Perfusion-driven Intravoxel Incoherent Motion (IVIM) MRI in Oncology: Applications, challenges, and future trends. Magn. Reson. Med. Sci..

[20] Postnikov E.B. (2020). Brownian yet non-Gaussian diffusion in heterogeneous media: From superstatistics to homogenization. New J. Phys..

[21] Cherstvy A.G., Metzler R. (2015). Ergodicity breaking and particle spreading in noisy heterogeneous diffusion processes. J. Chem. Phys..

[22] Kolmogorov A.N. (1941). Local structure of turbulence in an incompressible fluid at very high Reynolds numbers. Dokl. Akad. Nank. SSSR.

[23] Vannote R.L., Minshall G.W., Cummins K.W., Sedell J.R., Cushing C.E. (1980). The river Continuum concept. Can. J. Fish. Aquat. Sci..

